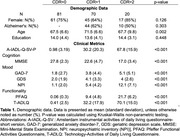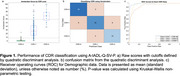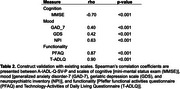# Adaptation and Validation of Amsterdam Instrumental Activities of Daily Living Questionnaire Short Version for Peru

**DOI:** 10.1002/alz70858_104928

**Published:** 2025-12-26

**Authors:** Gregory Brown, Diego Bustamante‐Paytan, Maria Fe Albujar‐Pereira, José Carlos Huilca, Katherine Agüero, Graciet Verastegui, Zadith Yauri, Pamela Bartolo, Daniela Bendezu, Rosa Montesinos, Nilton Custodio

**Affiliations:** ^1^ Instituto Peruano de Neurociencias, Lima, Lima, Peru; ^2^ University of California, San Francisco, San Francisco, CA, USA; ^3^ Equilibria, Lima, Lima, Peru; ^4^ Universidad de San Martín de Porres, Facultad de Medicina, Centro de Investigación del Envejecimiento, Lima, Lima, Peru; ^5^ Unidad de Investigación y Docencia, Equilibria, Lima, Peru., Lima, Lima, Peru

## Abstract

**Background:**

Instrumental Activities of Daily Living (IADL) are key indicators of functional decline in dementia, but culturally adapted tools for Latin America are scarce. The Amsterdam IADL Questionnaire Short Version (A‐IADL‐Q‐SV) has shown strong psychometric properties globally but has not been validated in Peru. This study aims to assess the validity and robustness of the Peruvian version of Amsterdam (A‐IADL‐Q‐SV‐P) in classifying dementia severity and its agreement with other clinical scales.

**Methods:**

A total of 171 participants were recruited a cognitive clinic in Peru. Clinical dementia rating scale (CDR) was used for dementia severity (81 CDR=0, 70 CDR=1, 20 CDR=2) Of the dementia patients (CDR^3^0), 54 had Alzheimer's disease and 36 had Frontotemporal dementia. Validity of the A‐IADL‐Q‐SV‐P was determined by ability to classify CDR level using quadratic discriminant analysis. The robustness of the scale was assessed by Spearman correlations with demographic variables (age, sex, and education). Further association with existing scales for cognitive [Mini‐Mental State Examination (MMSE)], mood [generalized anxiety disorder‐7 (GAD‐7), geriatric depression scale (GDS), and neuropsychiatric inventory (NPI)], and functionality [Pfeffer Functional Activities Questionnaire (PFAQ) and Technology‐Activities of Daily Living Questionnaire (T‐ADLQ)] status.

**Results:**

The A‐IADL‐Q‐SV‐P achieved excellent accuracy for classifying dementia severity (accuracy: 0.853, AUC>0.92). The ability to classify normal cognition had a sensitivity of 0.95 and a specificity of 0.91. These results were similar when investigating only the participants with either Alzheimer's disease or frontotemporal dementia (accuracy>0.88, sensitivity>0.95, and specificity>0.88, AUC>0.91). We also found weak associations with age, sex, and education (|rho|<0.2). Furthermore, the A‐IADL‐Q‐SV‐P had strong associations with other clinical scales (|rho|>0.4), particularly in relation to cognition (rho=‐0.70) and functionality (rho>0.87).

**Conclusion:**

A‐IADL‐Q‐SV‐P demonstrated excellent accuracy in classifying dementia severity, with strong correlations to cognitive and other functionality measures. The weak associations with demographic variables and validity in multiple dementia subtypes suggest robustness across diverse populations, supporting its utility as a culturally adapted tool for assessing functional decline in dementia.